# Exploring the *SiCCT* Gene Family and Its Role in Heading Date in Foxtail Millet

**DOI:** 10.3389/fpls.2022.863298

**Published:** 2022-06-09

**Authors:** Congcong Li, Jian Ma, Genping Wang, Haiquan Li, Hailong Wang, Guoliang Wang, Yanmiao Jiang, Yanan Liu, Guiming Liu, Guoqing Liu, Ruhong Cheng, Huan Wang, Jianhua Wei, Lei Yao

**Affiliations:** ^1^Beijing Academy of Agriculture and Forestry Sciences, Beijing, China; ^2^Institute of Biotechnology Research, Beijing Key Laboratory of Agricultural Genetic Resources and Biotechnology, Beijing, China; ^3^Biotechnology Research Institute, Chinese Academy of Agricultural Sciences, Beijing, China; ^4^Institute of Vegetable Research, Beijing Key Laboratory of Vegetable Germplasm Improvement, National Engineering Research Center for Vegetables, Beijing, China; ^5^Institute of Millet Crops, Hebei Academy of Agriculture and Forestry Sciences, Shijiazhuang, China

**Keywords:** gene duplication, transcriptional factor, CCT gene family, heading date, *SiPRR37*, foxtail millet

## Abstract

CCT transcription factors are involved in the regulation of photoperiod and abiotic stress in *Arabidopsis* and rice. It is not clear that how CCT gene family expand and regulate heading date in foxtail millet. In this study, we conducted a systematic analysis of the CCT gene family in foxtail millet. Thirty-nine CCT genes were identified and divided into four subfamilies based on functional motifs. Analysis showed that dispersed duplication played a predominant role in the expansion of CCT genes during evolution. Nucleotide diversity analysis suggested that genes in *CONSTANS* (*COL*)-like, *CCT MOTIF FAMILY* (*CMF*)-like, and pseudoresponse response regulator (PRR)-like subfamilies were subjected to selection. Fifteen CCT genes were colocalized with previous heading date quantitative trait loci (QTL) and genome-wide association analysis (GWAS) signals. Transgenic plants were then employed to confirm that overexpression of the CCT gene *SiPRR37* delayed the heading date and increased plant height. Our study first investigated the characterization and expansion of the CCT family in foxtail millet and demonstrated the role of *SiPRR37*. These results lay a significant foundation for further research on the function of CCT genes and provide a cue for the regulation of heading date.

## Introduction

Gene duplication is prevalent and supplies raw genetic material for evolution (Ohno, 1970; Zhang, 2003). There are multiple ways for genes to become duplicated, including unequal crossing over, retroposition, and chromosomal (or genome) duplication, which result in different consequences (Ramsey and Schemske, 1998; Zhang, 2003). As per the region’s size, duplications are classified as tandem, segmental duplication, retrotransposition, or whole-genome duplication (Vision et al., 2000; Simillion et al., 2002; Cannon et al., 2004). Polyploidy has been widely appreciated and acknowledged as a significant mechanism of adaptation and speciation, in part because polyploidy duplicates the members of the entire regulatory network (Ramsey and Schemske, 1998; Bowers et al., 2003; Shoemaker et al., 2006). In *Arabidopsis*\ *thaliana*, two or more rounds of polyploidy were identified by comparative genomic analysis (Ku et al., 2000). About 70 million years ago (mya), an ancient polyploidization event occurred before the divergence of the major cereals from one another, and a subsequent polyploid event happened over 11 mya in maize (Gaut et al., 2000; Paterson et al., 2004). The latest research indicates that the polyploidization in Poaceae occurred at ~96 mya, while the split of Andropogoneae (containing sorghum and maize) and Setaria occurred at ~51 mya (Wang et al., 2015). Compared to whole-genome duplication, segmental duplications are large, interspersed duplications that involve 1,000–200,000 nucleotides (Samonte and Eichler, 2002). Segmental duplication plays an essential role in promoting the generation of new resistance gene specificities in the plant (Cannon et al., 2004). In addition, segmentally duplicated genes may have divergent expression patterns; for example, two myb-homologous genes in maize, *p1* and *p2*, had a differential expression, generating tissue specificities (Zhang and Peterson, 2000). Tandem duplications usually result from unequal crossing-over; therefore, tandemly duplicated genes are linked in a chromosome (Zhang, 2003). As recent technology advances, genome structural variations, particularly copy-number variations (CNVs), have become the focus of attention. CNVs could be considered processes underlying gene duplication and loss, which tend to influence specific functional genes, such as environmental response-related genes (Korbel et al., 2008).

Under the model contributed to Ohno (1970), after duplication, one copy retains the ancestral function. In contrast, the other copies will be pseudogenized due to adverse selection or the acquisition of new tasks *via* the accumulation of substitutions. The subfunctionalization model and the duplication–degeneration–complementation model presume that two copies experienced degenerate mutations during evolution (Hughes, 1994; Force et al., 1999). Subneofunctionalization postulates rapid subfunctionalization accompanied by prolonged and substantial neofunctionalization in duplicated genes (He and Zhang, 2005). The above models have been demonstrated in specific organisms. Gene duplication contributes not only to genomic and organismal evolution but also to the evolution of a sophisticated gene network (Wagner, 1994; Zhang, 2003). Without gene duplication, genomic evolution would be limited. In addition, gene duplication has also been recognized as a mechanism of genome adaptation in the course of adaptation to stressful environments, such as the identification of CNVs in species adapting to novel conditions (Kondrashov, 2012).

The important switch in flowering plants is the transition from vegetative to reproductive development, which is regulated by multiple environmental and endogenous factors (Simpson and Dean, 2002). The CCT [CONSTANS (CO), CO-like, and TIMING OF CAB EXPRESSION1 (TOC1)] domain-containing protein family has been intensively studied in flowering plants, such as *A. thaliana*, rice, and maize, which mainly affect flowering time (Putterill et al., 1995; Strayer et al., 2000; Robson et al., 2001; Liu et al., 2020). In rice, a total of 41 CCT genes have been identified, 18 of which have been shown to regulate heading date (Zhang et al., 2015, 2021). *Hd1*, an orthologue gene of *CO* in rice, could promote the heading date under short-day conditions and function as an inhibitor under long-day conditions; *Hd1* accumulation was mediated by a RING-figure ubiquitin ligase (Yano et al., 2000; Yang et al., 2015). *TOC1* encodes a circadian clock-associated factor containing the pseudoresponse response regulator (PRR) domain apart from the CCT domain (Strayer et al., 2000). According to the functional domains, the CCT gene family in rice was divided into three subgroups: the *CCT MOTIF FAMILY* (*CMF*) subgroup, the *CONSTANS-like* (*COL*) subgroup, and the *PRR* subgroup, while another subgroup *TIFY* was also subdivided in maize (Cockram et al., 2012; Jin et al., 2018).

A thorough study of flowering time has been conducted in *Arabidopsis* and rice. The CCT genes play an important role in the molecular regulatory network of flowering time in plants. *FLOWERING LOCUST* (*FT*) is a molecular hub in flowering time pathways that encodes mobile florigen in leaves (Abe et al., 2005). The transcription of *FT* could be activated by *CO*, and the latter was activated by *Gigantea* (*GI*) in *Arabidopsis* under short-day conditions (Hayama et al., 2003; Hayama and Coupland, 2003). In addition, there was another signaling pathway with an early heading date 1 (*Ehd1*)-centered specific pathway under long-day conditions in monocots. *OsMADS50* activates the expression of *Ehd1*, and in turn, *Ehd1* activates *RFT1* (the long-day specific florigen) expression (Tsuji et al., 2011). In this pathway, an important CCT-motif gene, *Grains Height Date 7* (*Ghd7*), functions as a repressor of *Ehd1* to delay flowering (Xue et al., 2009). Many CCT family genes have been identified by QTL mapping and comparative genomic analysis in *Setaria*, such as the homologous genes of *OsPRR95* and *OsPRR59* (Mauro-Herrera et al., 2013).

Recently, map-based cloning and genome-wide association analysis (GWAS) have been utilized to perform genetic analysis of important traits in foxtail millet. Based on 916 accessions of foxtail millet, 13 signals related to heading date and early flowering were identified, including the homologous genes of known photoperiodic pathway genes *Heading Date 1* (*Hd1*) and *OsPRR37* (Jia et al., 2013). The homolog gene of *Hd1* in foxtail millet with a splicing variation from “GT” to “AT” was correlated with heading date, and *Hd1* was also under parallel domestication in rice, sorghum, and foxtail millet (Liu et al., 2015). Quantitative trait loci (QTL) mapping of 182 recombinant inbred lines (derived from a cross between foxtail millet accession B100 and green foxtail accession A10) under short-day (8 or 12 h light) and long-day (16 h light) conditions showed that the 8 and 12 h photoperiods shared more QTL, while 16 h light conditions only shared one QTL, which indicated that *Setaria* possessed secondary long-day genetic regulation apart from the short-day pathway (Doust et al., 2017). Another recombinant inbred line from a cross between foxtail millet accessions “Zhanggu” and “A2” also uncovered different QTLs under field conditions and short- and long-day photoperiods (Mauro-Herrera et al., 2013; Ni et al., 2017; Zhang et al., 2017). Although a significant number of QTLs have been identified by forward genetics, QTL intervals are still large, and only a few genes for heading date have been isolated in foxtail millet (Mauro-Herrera et al., 2013; Yoshitsu et al., 2017).

The high synteny and conservation of flowering time-related genes in plants provide insights into the regulatory mechanism of heading date in foxtail millet. The CCT family has been comprehensively analyzed in multiple plants, such as rice (Zhang et al., 2015, 2021), maize (Jin et al., 2018), wheat (Zheng et al., 2017), and soybean (Mengarelli and Zanor, 2021). However, the underlying expansion model and molecular mechanisms of CCT domain proteins remain largely unexplored in foxtail millet. Foxtail millet (*Setaria italica*) is an ancient cereal, whose attributes of small diploid genomes, self-fertilization, and strong abiotic stress accentuated foxtail millet as an ideal model for functional genomic studies (Doust et al., 2009; Li and Brutnell, 2011; Muthamilarasan and Prasad, 2015). Now, under pandemic, the foxtail millet has the potential to become a new staple crop (Muthamilarasan and Prasad, 2021). In this study, we identified and analyzed the molecular evolution and expansion patterns of the CCT family from the foxtail millet. The results showed that 15 CCT genes localized with the identified heading date QTLs. Combining genome-wide association analysis signals with the heading date, we verified the function of *SiPRR37* in the heading date regulation network. These results provide clues for governing the heading date and future genetic improvements.

## Materials and Methods

### Identification of CCT Family Genes in Foxtail Millet

The foxtail millet genome (version 2.2, Bennetzen et al., 2012) was downloaded from the plant genome database Phytozome.^1^ The Hidden Markov Model (HMM) program hmmsearch was applied to identify CCT proteins with the parameter “--cut_tc” to control model-specific thresholding. The HMMER profile of the CCT domain (PF06203) was downloaded from the PFAM protein family dataset (Finn et al., 2010).^2^ The basic properties of the CCT genes, namely, the length of amino acids, isoelectric point (PI), and molecular weights (MW), were estimated using the ExPASy program.^3^

### Phylogenetic Analysis and Structure of CCT Proteins

To analyze the evolutionary relationship, the reference genome sequences of *Setaria viridis* (V2.1), *Sorghum bicolor* (V3.1.1), *Oryza sativa* (V7.0), and *Zea mays* (RefGen V4) were downloaded from Phytozome. Similarly, we identified the putative CCT genes in green foxtail, rice, maize, and sorghum by HMM profiles corresponding to the CCT domain. The primary protein of CCT family from these four species was aligned with clustalW and the phylogenetic tree was constructed by MEGA-X using the neighbor-joining (NJ) method with 1,000 bootstraps (Kumar et al., 2018). Finally, the evolutionary tree was visualized and illuminated by the software iTOL (Letunic and Bork, 2007).^4^ The conserved motifs of CCT proteins in foxtail millet were determined using the Pfam 33.1 database. Promoter sequences (2 kb upstream of the transcription start site) were extracted based on the genome sequence and annotation file, and promoter cis-acting elements were identified by Plantcare.^5^ The Gene Structure Display Service (GSDS 2.0; Hu et al., 2015) was used to draw the main protein domain and cis-regulatory elements distribution.

### Chromosomal Localization and Duplication of CCT Family Genes

Chromosomal localization information for CCT genes in foxtail millet was identified from the reference genome database, and the genes were mapped to nine chromosomes using MapChart software according to the manual (Voorrips, 2002).^6^ To investigate the evolutional mechanism of the CCT family genes, five modes of duplication as whole-genome duplicates (WGD), tandem duplicates (TD), proximal duplicates (less than 10 gene distance on the same chromosome: PD), transposed duplicates (transposed gene duplications: TRD), or dispersed duplicates (DSD) occurred in CCT genes were obtained from PlantDGD database (Qiao et al., 2019).^7^ Gene duplication and synteny analysis were also performed using MCScanX software (Wang et al., 2012). The whole-genome duplication pattern was visualized using Circos software (Krzywinski et al., 2009).

### Expression Patterns and Alternative Splicing Event Identification of CCT Family Genes

The expression profile of some CCT genes in foxtail millet cultivar “Jingu 21” was extracted from the multi-omics database for *S. italica* (MDSi; Yang et al., 2020),^8^ which contained 23 samples from several tissues (including seeds, leaf, panicle, stem, and root) at vegetative and reproductive stages. The expression data (fragments per kilobase of transcript per million fragments mapped, FPKM values) were normalized into log2 (1 + FPKM), and the heat map was constructed using the R package “pheatmap” (version 1.0.12). To identify alternative splicing (AS) event, the genomic annotation file of the foxtail millet was used to extract all transcripts of CCT family genes. Then, AS events identification was performed by using ASTALAVISTA (Foissac and Sammeth, 2007).

### Variation Distribution of CCTs and Nucleotide Diversity

To characterize the natural variation of CCT genes in foxtail millet, single-nucleotide polymorphisms (SNPs) in the coding region of CCT genes were identified based on the haplotype variation map from whole-genome resequencing of 312 accessions (Li et al., 2021). In addition, we extracted the SNPs from the genomic sequence, which referred to the sequence from the transcription initiation site to the transcription stop site. SNPs from the promoter region (within 2 kb upstream of the transcription start site) were also extracted. The average number of nucleotide differences per site between the two random sequences (π), the Watterson estimator (θ), and Tajima’s D were calculated using DnaSP v5.0 according to the manual (Librado and Rozas, 2009). The π ratio of genetic diversity in landraces to that in improved cultivars of foxtail millet was calculated for selection signals related to screening improvement.

### Functional Identification of *SiPRR37* by Transformation

We previously found that *SiPRR37* had different haplotypes with or without the *Tc1-Mariner* transposon (Li et al., 2021). To validate the function of the CCT gene *SiPRR37*, we cloned the functional *SiPRR37* haplotype and performed overexpression assays to generate the OE-SiPRR37 transgenic lines. Total RNA was extracted from the leaf tissue of foxtail millet landrace “Daqingmiaoyuci” using an RNAprep Prue plant kit (Tiangen Biotech Co., Ltd.). After isolation, first-strand cDNA synthesis was performed using the Transcriptor First Strand cDNA synthesis kit (Takara Biotech Co., Ltd) according to the manufacturer’s protocol. The 2.2-kb coding sequence of *SiPRR37* was amplified using KOD FX (TOYOBO Life Science) and cloned into the binary vector pCUbi1390 driven by the ubiquitin promoter. The specific primers were designed using Primer-BLAST (National Center for Biotechnology Information, Maryland, United States) and are listed in Supplementary Table S7. The resultant plasmid was transformed into *Agrobacterium tumefaciens* strain EHA105. *Agrobacterium*-mediated *ProUbi::SiPRR37* transformation was performed according to a previous study with some modifications (Yang et al., 2020). First, the palea and lemma of Ci846 mature seeds were removed and surface sterilized. The rinsed seeds were cultured on a medium for callus induction at 28°C in the dark for 8–10 weeks. Strain EHA105 harboring the *ProUbi::SiPRR37* vector was cultured overnight and resuspended to a density of 600 nm (OD_600_) = 0.5. High-quality calli were infected in resuspended cells for 5 min and transferred into a co-cultivation medium at 22°C in the dark. Then, calli were transferred to callus induction medium and selection medium with hygromycin B successively. Resistant calli were transferred to the shoot induction medium and cultured for root formation. Putative transgenic plants with healthy roots were moved to pots and detected by PCR. In addition, the expression level of positive transgenic plants was measured by qPCR.

The T_2_ transgenic lines with a higher expression level of *SiPRR37* were chosen to observe the phenotype change. Wild-type Ci846 and overexpressing transgenic lines were grown under field conditions in Beijing (40°13′N, 116°13′E). Heading date was scored by means ± SDs (*n* = 20) in the field conditions.

### Subcellular Localization and Transcriptional Activity Assay

To examine the subcellular localization, *Agrobacterium* GV3101 containing pCAMBIA1305-GFP ligated to two haplotypes of *SiPRR37* was transformed into *Nicotiana benthamiana* leaves. After 2 days of incubation, the infected leaves were harvested and used to observe GFP fluorescence using a confocal laser scanning microscope Nikon A1R. For the transcriptional activity assay, the functional and mutational SiPRR37 protein was amplified and cloned into the GAL4BD vector. The plasmids containing GAL4BD-SiPRR37/GAL4BD-Siprr37, pRTL, and 35sLUC were transformed into *Arabidopsis* protoplast cells as previously described with modifications (Yoo et al., 2007). The positive and negative controls were GAL4BD-VP16 and GAL4BD, respectively. Due to the transcriptional inhibitory activity of *SiPRR37*, the CDS of *SiPRR37* and *Siprr37* was cloned and introduced into GAL4BD-VP16. After incubation in the dark overnight, the protoplast cells were harvested and used to measure luciferase activity according to the manufacturer’s instructions for the Dual-Luciferase Reporter (DLRTM) Assay System (Promega).

### RNA Sequencing

RNA sequencing was performed on T_2_ OE-SiPRR37 plants under long-day conditions. The leaf tissues with three biological replicates were harvested at the vegetative stage for T_2_ plants. Six samples were prepared for 150 bp paired-end sequencing using an Illumina HiSeq X Ten platform. The raw data were trimmed and mapped to the foxtail millet reference genome using HISAT2 (hierarchical indexing for spliced alignment of transcripts; Kim et al., 2015). The FPKM of all genes was calculated using StringTie (v2.1.4, Pertea et al., 2015). Differentially expressed genes (DEGs) were detected using the R package edgeR (Robinson et al., 2010) following the criteria [|log_2_(fold-change)| > 1 and false discovery rate < 0.01]. The DEGs were validated through qRT-PCR. MapMan bins of the foxtail millet genome were assigned (Thimm et al., 2004).^9^ Open-source MapMan software was used to identify the categories of differentially expressed genes. The RNA-seq raw data were submitted to the National Center for Biotechnology Information (NCBI) Bioproject under accession number PRJNA797949.

## Results

### Identification and Phylogenetic Analysis of CCT Proteins in Foxtail Millet

A total of 39 putative CCT genes were identified in the *S*. *italic* reference genome (Supplementary Table S1). The amino acid number of these CCT proteins varied greatly, ranging from 122 (*Seita*.*8G159000*) to 760 (*Seita*.*9G445200*), and the molecular weight ranged from 13.82 to 82.67 kDa. CCT family genes were distributed on each chromosome (Supplementary Figure S1). There were nine CCT genes on chromosome 9; chromosomes 1 and 4 harbored seven, and five CCT genes, respectively; chromosomes 2 and 3 each contained four CCT genes; chromosomes 6, 7, and 8 each contained three CCT genes; and chromosome 5 carried only one CCT gene.

**Figure 1:**
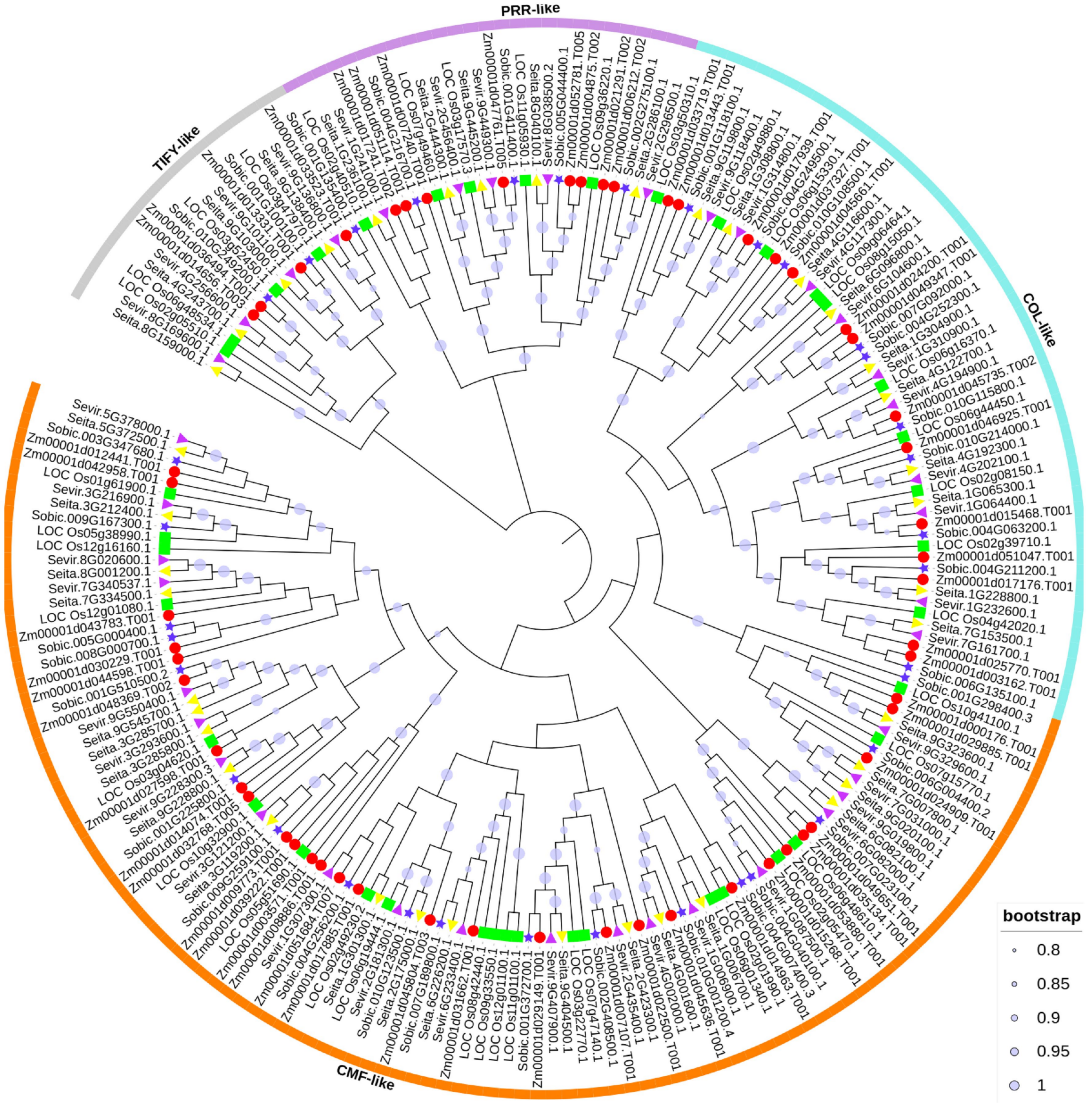
Phylogenetic relationship of CCT family genes. The putative CCT genes from rice, maize, sorghum, green foxtail, and foxtail millet were identified to construct the neighbor-joining phylogenetic tree. Solid lines with different colors enclose predominantly *CONSTANS* (COL)-like, *CCT MOTIF FAMILY* (CMF)-like, pseudoresponse response regulator (PRR)-like, and TIFY-like subfamilies. To distinguish the species, the red circle, blue pentagram, green square, purple triangle, and yellow triangle represent the CCT proteins from maize, sorghum, rice, green foxtail and foxtail millet, respectively.

To investigate the evolutionary relationship of CCT proteins in *S. viridis, S*. *italic*, *O. sativa*, *S. bicolor*, and *Z. mays*, a phylogenetic tree was constructed. A total of 208 CCT genes were identified from five Poaceae plants, with 39 CCT genes in foxtail millet, 39 CCT genes in green foxtail, 40 CCT genes in rice, 35 CCT genes in sorghum, and 55 CCT genes in maize. The number of genes in rice and maize was similar to previous studies (Figure 1; Zhang et al., 2015; Jin et al., 2018). Based on the domains they contained, all CCT genes were also divided into four subfamilies, with the largest CMF-like subfamily containing 108 members. The COL-like subfamily was composed of 54 members, the PRR-like subfamily had 27 members, and the TIFY-like subfamily involved 19 members. In each subfamily, CCT genes from the five species were closely clustered, suggesting that CCT gene families may have evolved before species differentiation.

**Figure 2:**
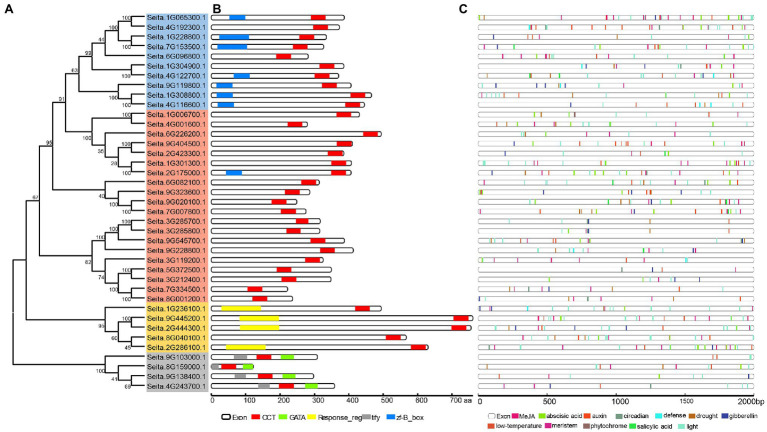
Phylogenetic relationship, conserved motifs, and promoter regulatory element analysis of foxtail millet CCT genes. **(A)** The full-length protein sequences of CCT genes were used to construct the neighbor-joining phylogenetic tree with 1,000 bootstrap replicates. The blue, orange, yellow, and gray shadow color indicated the COL-like, CMF-like, PRR-like, and TIFY-like subfamilies, respectively. **(B)** The main motif composition of CCT proteins is shown. Each motif is represented in a colored box. Protein length is scaled at the bottom. **(C)** The main response elements in the promoters are shown. Different regulatory elements are displayed in different colors.

Based on the topology of CCT genes in four Poaceae plants, there were 10 COL-like genes, 20 CMF-like genes, five PRR-like genes, and four TIFY-like genes in foxtail millet (Figure 2A). The analysis of these CCT protein structures showed a similar pattern with the CCT family divergence (Figure 2B). It also indicated that different subfamilies encoded proteins of various lengths and that the PRR subfamily was the longest. In addition, four genes only including the CCT domain were clustered in the COL subfamily or PRR subfamily, and one gene with zf-B box domain was clustered in the CMF-like subfamily. Partial CCT genes also exhibited a similar phenomenon in maize (Jin et al., 2018). It could be inferred that these genes lost the functional B-box type zinc finger domain during CCT gene family expansion.

The promoter sequences of CCT genes were analyzed in the PlantCARE database. The result showed that the promoters contained over 20 conserved elements, such as G-box, sp1, and TGA-element, with the highest number of G-box and ABRE elements (Figure 2C; Supplementary Table S2). These conserved elements were related to light responsiveness, plant hormones (abscisic acid, gibberellin, MeJA, and salicylic acid), and stress (low temperature and drought). Almost each CCT gene included a light responsiveness element, which inferred that the CCT domain genes were related to photoperiod.

### Expression Profiles and AS Events of CCT Family Genes

To further study the functions of CCT genes, we detected the expression profile of partial CCT genes in different tissues and multiple growth stages. Overall, CCT genes in the same subfamily displayed similar expression patterns (Supplementary Figure S2). For example, most of the CMF subfamily genes were not expressed or had a lower expression level in all 23 tissues/stages, while the PRR subfamily genes showed a higher expression level. Moreover, we compared the expression patterns of segmentally duplicated genes. Some duplicated genes, including *Seita*.*1G006700*/*Seita*.*4G001600*, had similar expression patterns, while largely duplicated genes exhibited different patterns in most tissues. It was speculated that the newly duplicated genes played a powerful role in functional innovation.

In total, 11 AS events were identified in the CCT family genes, which were mainly distributed in six genes containing two CMF-like subfamily genes, two PRR-like subfamily genes, one COL-like gene, and TIFY-like subfamily gene (Table 1). Among of six AS genes, *Seita.2G444300* and *Seita.1G308800* consisted of two types of AS events, while AS events did not occur in their highly homologous genes.

**Table 1:** Statistics of alternative splicing events in foxtail millet CCT genes.

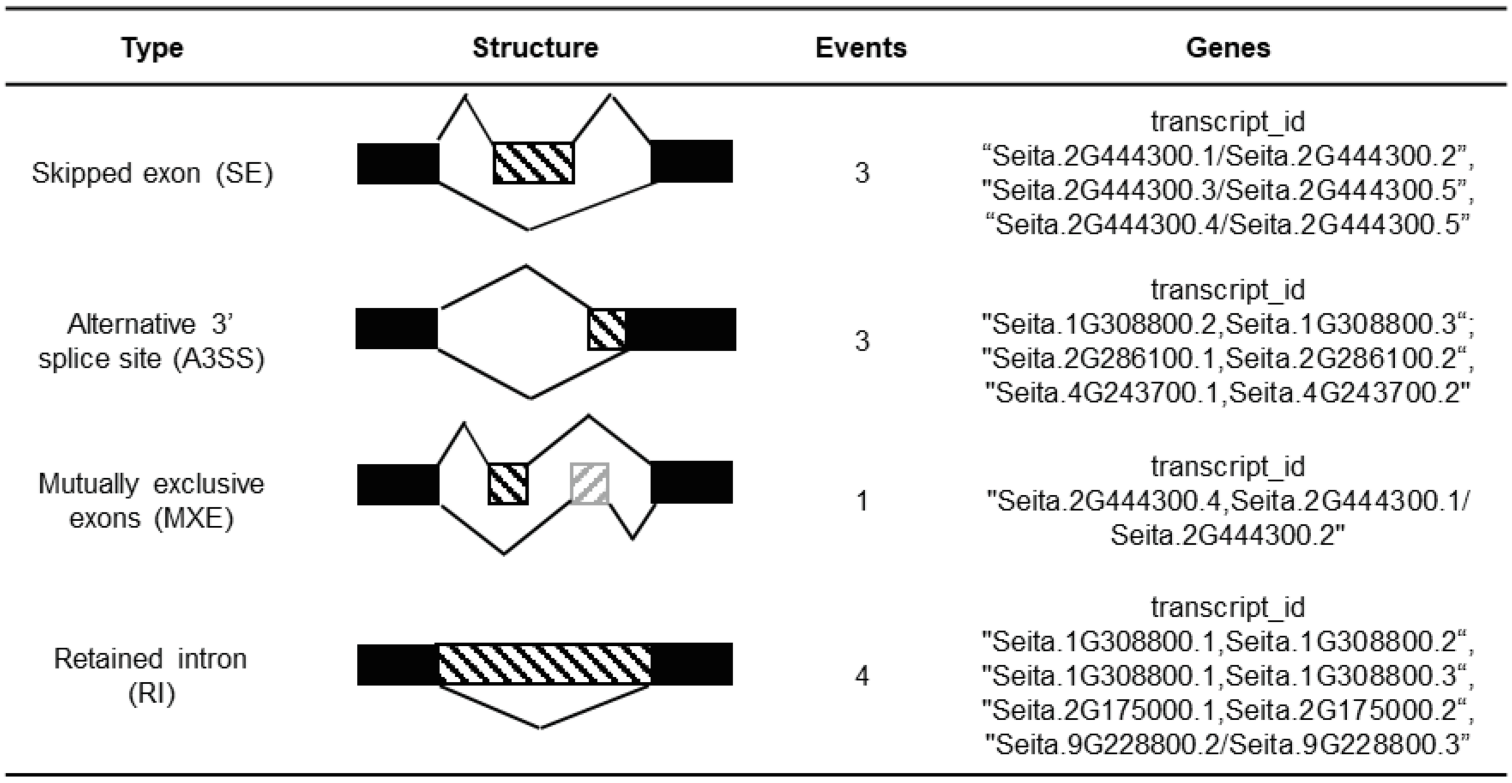

### Gene Duplication of CCT Family Genes

**Figure 3:**
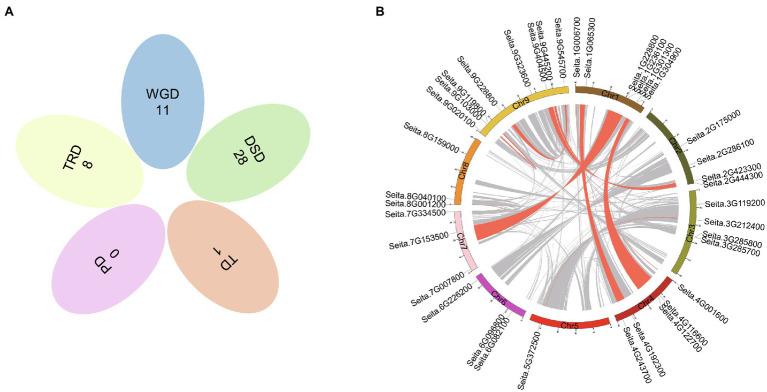
Possible modes of duplications in foxtail millet CCT genes. **(A)** Venn diagram shows the possible modes of duplication. WGD, whole-genome duplicates; DSD, dispersed duplicates; TRD, transposed duplicates; PD, proximal duplicates; and TD, tandem duplicates. **(B)** Circos plot showing the genomic distribution of CCT genes and the synteny blocks (gray lines) in foxtail millet. Red lines highlight the synteny blocks, including duplicated pairs of CCT genes.

Gene duplication is an important way for gene family expansion. To investigate the mode of duplication, we searched the PlantDGD database to extract all kinds of duplicates in foxtail millet CCT family genes. The results showed that a total of 48 duplication events occurred, including 11 WGD, 8 TRD, 28 DSD, and only one pair of tandem duplication genes, *Seita*.*3G285700* and *Seita*.*3G285800*, closely clustering on chromosome 3 (Figure 3A; Supplementary Figure S1).

Eleven WGD events were identified, including four duplication events in the COL-like subfamily, five events in the CMF-like subfamily, one event in the PRR subfamily, and one in the TIFY subfamily (Figure 3B). Interestingly, four WGD events (*Seita*.*1G006700*/ *Seita*.*4G001600*, *Seita*.*1G065300*/*Seita*.*4G192300*, *Seita*.*1G304900*/*Seita*.*4G122700*, and *Seita*.*1G308800*/*Seita*.*4G116600*) arose between chromosomes 1 and 4 (Figure 3B; Supplementary Table S3). Among eight TRD events, we found that the transposon jumping near *Seita.2G444300* was highly active, which was associated with the other two genes (*Seita.1G236100* and *Seita.2G286100*). The intricate network of DSD events played a predominant role in CCT gene family expansion (Supplementary Table S3). The result indicated that DSD events mainly happened in the same subfamily. And most genes participated in two or three rounds of dispersed duplication events. Extensive duplication facilitates the rapid evolution of the CCT gene family.

### Nucleotide Diversity of CCT Family Genes in Foxtail Millet

In an attempt to characterize the nucleotide diversity of CCT genes, we analyzed the genomic region and the promoter region of CCT family genes within 203 foxtail millet landraces and 96 improved cultivars. The results showed that the average nucleotide diversity of each CCT subfamily was higher than that of the whole genome in foxtail millet (π = 1.621 × 10^−3^ in traditional landraces and π = 1.568 × 10^−3^ in improved cultivars; Li et al., 2021; Table 2). In the genomic region, the TIFY subfamily showed lower nucleotide diversity (π = 2.01 × 10^−3^) than that of the other three subfamilies. Overall, the nucleotide diversity in the promoter regions exhibited a similar level to the genomic regions, except for the TIFY subfamily (Table 2). In addition, Tajima’s D of three subfamilies except for the TIFY subfamily reached a significant positive level. Interestingly, we also found that the diversity of the COL and TIFY subfamilies was lower in improved cultivars than in landraces, while the opposite pattern appeared in the CMF family. Therefore, four subfamilies underwent selection to different extents during improvement.

**Table 2:** Nucleotide diversity of CCT family genes in landraces and improved cultivars.

	Promoter			Genomic DNA		
	π (×10^−3^)	θ (×10^−3^)	Tajima’s D	π (×10^−3^)	θ (×10^−3^)	Tajima’s D
**COL-like**						
All	3.18	1.58	3.02^**^	3.38	1.58	3.42^**^
Landraces	3.42	1.69	3.13^**^	3.49	1.69	3.28^**^
Improved cultivars	2.45	1.93	0.89	2.85	1.95	1.53
**CMF-like**						
All	3.03	1.58	2.69^*^	3.21	1.47	3.41^**^
Landraces	2.80	1.70	1.96	3.04	1.58	2.76^*^
Improved cultivars	3.41	1.77	3.05^**^	3.52	1.76	3.23^**^
**PRR-like**						
All	3.52	1.58	3.48^**^	3.36	1.58	3.35^**^
Landraces	3.57	1.70	3.26^**^	2.46	1.70	3.19^**^
Improved cultivars	3.10	1.95	1.89	2.68	1.95	1.24
**TIFY-like**						
All	2.79	1.58	2.01	2.01	1.58	0.76
Landraces	3.00	1.70	2.14^*^	2.24	1.70	0.92
Improved cultivars	2.28	1.87	0.65	1.39	1.35	0.08

* represents *p* < 0.05;

** represents *p* < 0.01.

Based on the haplotype map of 312 accessions in foxtail millet, we cataloged the SNP variation profile of 39 CCT genes. The results showed that a total of 19 CCT genes had a missense mutation, including four CMF-like genes, nine COL-like genes, three PRR-like genes, and three TIFY-like genes (Supplementary Table S4). Population genetic variation provides the rich potential to facilitate adaptation to novel environments. To determine whether the CCT gene family could be associated with improvement selection, we utilized π to screen the selective signals. We found only one TIFY-like gene (*Seita*.*9G103000*) located in the improvement-related selective interval (Supplementary Figure S3).

### Comparison of CCT Gene Localization and Heading Date QTLs

The protein family containing the CCT domain is mainly related to the flowering time pathway. To investigate the association between the CCT genes and heading date in foxtail millet, we collected heading date QTLs and GWAS signals according to previous studies and compared CCT gene localization with known heading date QTLs (Jia et al., 2013; Mauro-Herrera et al., 2013; Doust et al., 2017; Zhang et al., 2017; Li et al., 2021). Twenty-seven heading date-related QTLs and 40 GWAS signals were identified (Supplementary Tables S5, S6). Among 39 CCT family genes, 15 genes were located in the QTL regions or adjacent to the GWAS signals, including five COL-like genes, seven CMF-like genes, and three PRR-like genes (Supplementary Tables S5, S6). These genes may play an important role in regulating heading date.

### *SiPRR37* Is Related to Foxtail Millet Heading Date

**Figure 4:**
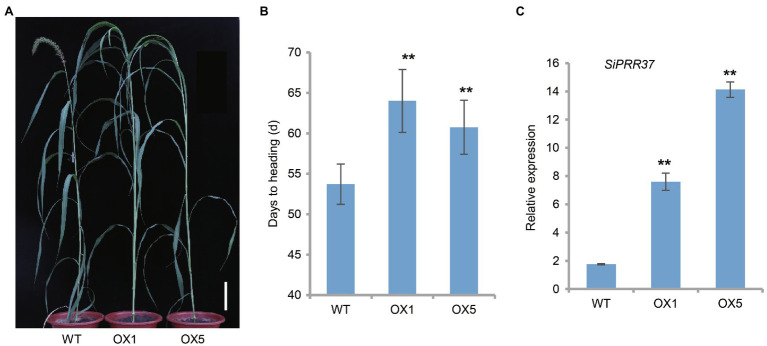
Phenotypical characterization of transgenic plants overexpressing the *SiPRR37* gene in foxtail millet. **(A)** Representative graphic of the overexpressing transgenic plants at the heading date stage grown in the Beijing field in 2020. The scale bar indicates 10 cm. **(B)** Statistical analysis of days to heading of transgenic lines and wild type by Student’s t-test. Error bars represent ± SD (*n* = 10). ^**^ indicates *p* < 0.01. **(C)** The transcriptional levels of SiPRR37 in the overexpressing plants and wild type. Data were shown as means ± SD (*n* = 3).

In a previous study, we identified a significant association interval including *SiPRR37* by GWAS (Li et al., 2021). *SiPRR37* belongs to a member of the CCT transcription factor family. To validate the function of *SiPRR37*, we introduced the functional haplotype into the robust transformation receptor Ci846. The presence of the overexpression cassette in the transgenic lines was confirmed by PCR analysis and sequencing of the PCR fragment. Significantly enhanced expression of *SiPRR37* in transgenic lines was also confirmed by real-time RT-PCR (Figure 4C). The positive transgenic lines showed an obvious difference in heading date compared with wild-type plants in the T1 generation under field conditions (Figure 4A). The SiPRR37-OE lines headed about 10 days later than the wild-type plant, indicating that *SiPRR37* negatively regulated the heading date in foxtail millet (Figure 4B).

It was still unclear whether the SiPRR37-OE lines delayed the heading date under different conditions. To address this question, we planted the transgenic lines on June 22 and observed the phenotype in the greenhouse with a normal long-day photoperiod. We found that the transgenic line OX5 suppressed heading compared to the wild type (Supplementary Figure S4B). In addition, the SiPRR37-OE line OX5 influenced plant height simultaneously (Supplementary Figures S4A,C). Taken together, these results suggest that *SiPRR37* is an important gene underlying heading date and plant architecture in foxtail millet.

*SiPRR37* had two main haplotypes in foxtail millet of 312 accessions. In the mutant, transcription was truncated, and the CCT domain was lost due to *Tc1-Marina* transposon insertion (Li et al., 2021). Thus, we observed the sublocalization of two haplotypes. The two haplotypes (*SiPRR37* and *Siprr37*) with GFP labels driven by the CaMV35S promoter were introduced into tobacco leaves. The SiPRR37-GFP signal appeared in the nucleus, while the Siprr37-GFP signal was detected in the nucleus and cytoplasm, similar to the localization of the blank control (Supplementary Figure S5A). These results validate that the CCT domain plays an important role in the nuclear localization of proteins (Robson et al., 2001). Thus, we evaluated the transcriptional activity of two haplotypes in *Arabidopsis* protoplasts. The results showed that *SiPRR37* exhibited transcriptional inhibitory activity, which indicated that *SiPRR37* functioned as a repressor in regulating heading date (Supplementary Figure S5B). In contrast, *Siprr37* lost its transcriptional activity despite its nuclear localization.

**Figure 5:**
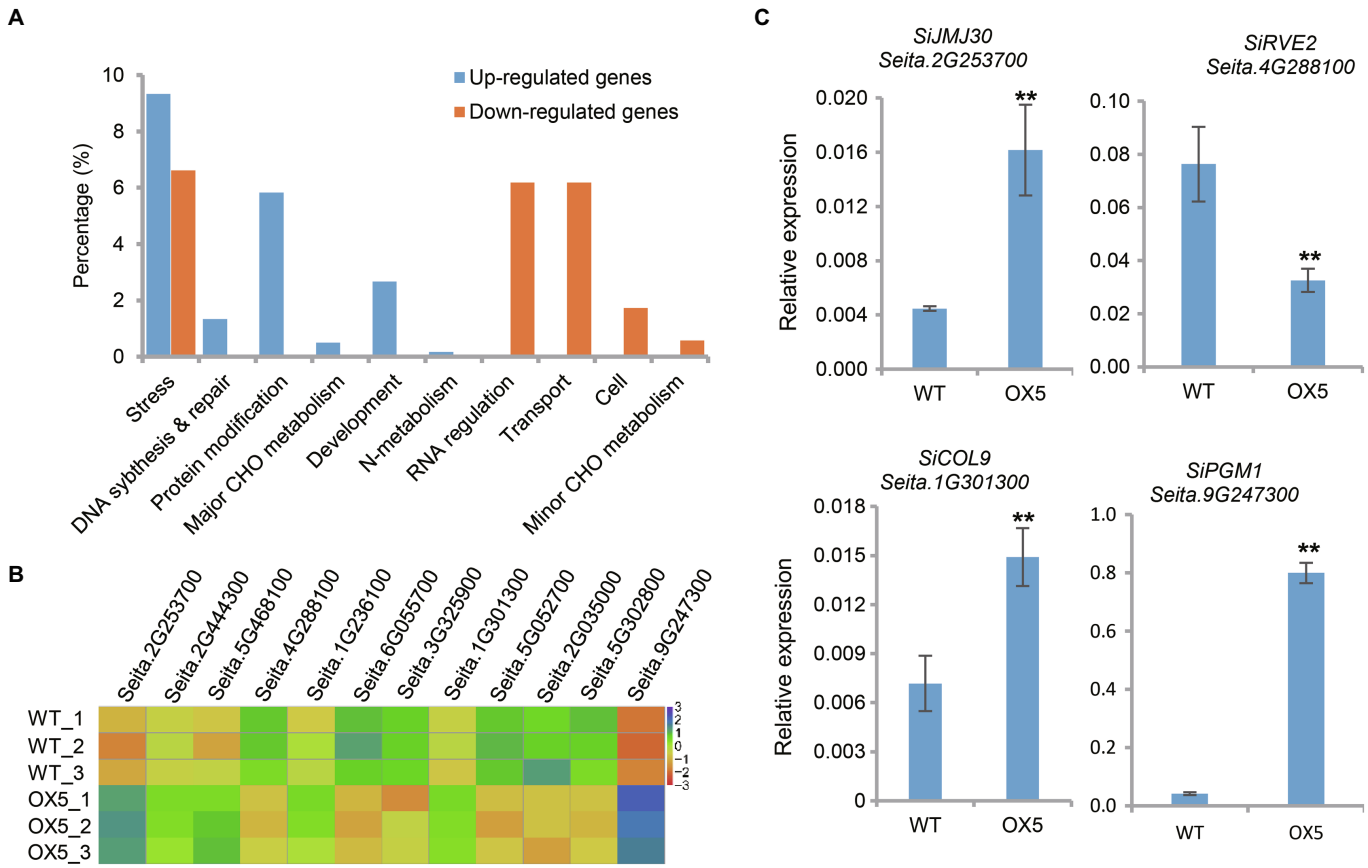
Differentially expressed genes (DEGs) analysis between overexpression transgenic plants OX5 and wild type. **(A)** Functional classification of DEGs according to Mapman bin categories. Upregulated and downregulated DEGs were displayed in blue and orange, respectively. **(B)** The heat map of 12 DEGs between wild type and OX5. These DEGs were homologous genes of known flowering-related genes in Arabidopsis. **(C)** The relative expression of partial DEGs was confirmed using real-time qPCR. The asterisks indicate a statistically significant difference (^**^p < 0.01, Student test).

To study the effect of *SiPRR37* on the downstream genes, RNA-seq was performed on the leaves of the wild type and SiPRR37-OE transgenic line OX5 at the vegetative stage. Compared with the wild-type line, the SiPRR37-OE transgenic line has 1,295 DEGs, including 695 downregulated genes and 600 upregulated genes. DEGs were significantly enriched in MapMan categories, including signal, stress, protein, secondary metabolism, nucleotide metabolism, and cofactor metabolism, and the downregulated and upregulated gene compositions were different (Figure 5A). Among 695 downregulated genes, the most prominent subcategory was transcriptional regulation, which contained 36 transcription factors referring to the diverse aspects of development. In addition, an abundance of DEGs involved stress, which was also the target gene of *PRR7* in *Arabidopsis* (Liu et al., 2013). This result indicated that *SiPRR37* may participate in multiple regulation pathways besides heading date.

Combined with the known flowering time genes from *Arabidopsis*, 12 DEGs were identified by comparative analysis, which were involved in the circadian clock, photoperiodism, sugar, and general pathways (Figure 5B). *SiPRR37* (*Seita*.*2G444300*) is a circadian clock-related gene whose overexpression influenced the expression of other circadian clock-related genes, such as *Seita*.*6G055700* (*SiLHY*, the homolog of *LATE ELONGATED HYPOCOTYL*), *Seita*.*1G236100* (*SiTOC1*, the homolog of *TIMING OF CAB EXPRESSION 1*), *Seita*.*4G288100* (*SiRVE2*, the homolog of *REVEILLE 2*), and *Seita*.*5G468100* (*SiPCL1*, the homolog of *PHYTOCLOCK 1*). The expression profile of the clock genes demonstrated that *Arabidopsis PRR7* can inhibit the expression of *LHY*, while *LHY* can bind to and activate the expression of PRR7 and PRR9 (Salomé et al., 2010). Notably, multiple photoperiodic genes were downregulated, including *Seita*.*3G325900* (the homolog of *AS1*, *ASYMMETRIC LEAVES 1*), *Seita*.*5G052700* (the homolog of *CDF2*, *CYCLING DOF FACTOR 2*), *Seita*.*2G035000* (*LATE*, *LATE FLOWERING*), and *Seita*.*5G302800* (the homolog of *SPA3*, *SPA1-RELATED 3*). *Seita*.*1G301300* (the homolog of *COL9*, *CONSTANS-LIKE 9*) was upregulated. One glycometabolism-related gene, *Seita*.*9G247300* (the homolog of *PGM1*, *PHOSPHOGLUCOMUTASE*), was also significantly upregulated. We also found two other DEGs belonging to the CCT family, *Seita*.*2G175000* (the homolog of *OsCCT22*; Zhang et al., 2021) and *Seita*.*9G228800* (a member of the CMF subfamily), were also upregulated. The qRT-PCR results showed that the expression patterns of most genes were consistent with the RNA-seq results (Figure 5C).

## Discussion

### Conservation and Divergence of CCT Family Genes

The important feature of the CCT family is the 43–45 conserved amino acids in the carboxy terminus, which could possess the function of a nuclear localization signal (Strayer et al., 2000; Robson et al., 2001). The CCT domain proteins participate in the signal cascade of environmental factors, such as light, gravity, and temperature, which may be involved in the regulation of the photoperiod pathway, circadian clock, and abiotic responses (Putterill et al., 1995; Kaczorowski and Quail, 2003; Salomé and McClung, 2005). In maize, the CCT genes can be classified into four subfamilies corresponding to the encoded domains, namely, the COL subfamily, the CMF subfamily, the PRR subfamily, and the TIFY subfamily (Jin et al., 2018). The COL-like genes possessed CCT and B-box zinc finger domains. The CMF genes only had a CCT domain. The PRR genes had CCT and response regulator receiver domains, while the TIFY genes held CCT, TIFY, and GATA zinc finger domains.

To date, at least 18 CCT domain-containing genes in rice have been isolated that participate in regulating heading date (Zhang et al., 2021). *Hd1* was the first CCT family gene to be identified, and its expression reached a peak during the night under long- and short-day conditions (Yano et al., 2000; Hayama et al., 2003). Apart from *Hd1*, the other three genes (*Ghd7*, *Ghd7*.*1/OsPRR37*, and *DTH2*) were also obtained by a map-based cloning strategy. *DTH2* (*days to heading on chromosome 2*) encodes a COL-like protein, which enhances adaptation for the northward expansion of rice cultivation (Wu et al., 2013). *Ghd7* belongs to the CMF-like subfamily, while *Ghd7*.*1/OsPRR37* belongs to the PRR-like subfamily, and both genes could cause pleiotropic effects, including delaying heading date, increasing plant height, and grain number per panicle (Xue et al., 2009; Yan et al., 2013; Gao et al., 2014). Other CCT genes, for example, *OsCCT01*, *OsCCT03*, and *OsCCT41* were obtained through reverse genetics and functioned as repressors or activators in the flowering regulation network (Zhang et al., 2015, 2021). In addition, multiple genes are involved in abiotic stress responses. For example, overexpression of *Ghd7* increased drought sensitivity, and *Ghd7* could strongly respond to abscisic acid, jasmonic acid, and high-temperature stress (Weng et al., 2014). In *Arabidopsis*, *AtCOL4* also participates in stress to salt through the ABA-dependent signaling pathway (Min et al., 2015).

In this study, a total of 39 genes of the CCT family, including CMF-like, COL-like, PRR-like, and TIFY-like subfamilies, were identified in foxtail millet. Among the different species, genes of the corresponding subfamily were clustered. This further proved that the CCT domain genes evolved before species divergence (Cockram et al., 2012). For example, genes *OsCOL4*, *OsCOL10*, and *OsCOL9* belong to the COL subfamily, and the overexpression of *OsCOL4* or *OsCOL10* in rice delays heading date regardless of day length, while the overexpression of *OsCOL9* also enhances rice blast resistance by interacting with the receptor for activated C-kinase 1 (Lee et al., 2010; Liu et al., 2016; Tan et al., 2016). In addition, the expression pattern of partially homologous genes in different tissues also exhibited vast diversity (Supplementary Figure S2).

Comparative genome analysis showed that the genes among foxtail millet, sorghum, and maize were highly conserved and had a higher identity in the panicoids (Bennetzen et al., 2012; Zhang et al., 2012). Considering that the CCT family genes evolved before the divergence between the monocots and dicots (Cockram et al., 2012), we speculated that the CCT genes in cereal crops possessed a conserved function. Thus, it will provide a reference for functional genome research of CCT genes in foxtail millet.

### Expansion of the CCT Gene Family in Foxtail Millet

In this study, we comprehensively analyzed the expansion mode of the CCT gene family in foxtail millet, which has not been performed in maize (Jin et al., 2018), rice (Zhang et al., 2015, 2021), wheat (Zheng et al., 2017), and soybean (Mengarelli and Zanor, 2021). A large number of duplicated genes occurred after the pan-grass polyploidization ~96 mya (Wang et al., 2015). Until now, 11 whole-genome duplicated CCT gene pairs are still identified in the foxtail millet genome (Figure 3A), including genes from CMF-like, COL-like, PRR-like, and TIFY-like subfamilies, which further inferred CCT gene family evolved before Poaceae species differentiation. Among these WGD events, four duplication events took place between Chr1 and Chr 4, two happened between Chr2 and Chr 9, one occurred between Chr1 and Chr 7, and one appeared between Chr3 and Chr 5, which was consistent with long synteny blocks in foxtail millet genome (Zhang et al., 2012).

We identified 48 duplication events that were classified into four categories: 11 WGD (22.9%), 28 DSD (58.3%), 8 TRD (2.1%), 1 TD (16.7%; Figure 3A). Our result suggested that DSD significantly contributed to CCT gene family expansion in foxtail millet. The duplication modes in different gene families or organisms may be discrepant. For example, the tandem duplication was the primary driving force for the expansion of the *BpUGT* gene family in *Broussonetia papyrifera* (Wang et al., 2021). While the dispersed duplication could directly affect the functional diversification by asymmetric regulatory divergence (Owens et al., 2013). Besides, alternative splicing is also an important regulatory mechanism for modulating gene expression and functional diversity (Lareau et al., 2004). However, AS events were only identified in six CCT genes (Table 1). One of the reasons may be that WGD-derived duplicated genes decrease the AS frequency (Shen et al., 2014). Therefore, the gene family could expand through duplication, while the duplicated genes maybe diverge over time to perform different functions.

### *SiPRR37* May Confer Diverse Functions

*SiPRR37*, which is the homologous gene of *PRR7* in *Arabidopsis*, encodes a circadian rhythm protein. Two allelic mutants of *prr7* exhibited reduced sensitivity to both continuous red and far-red light, and the stability of the PRR7 protein was regulated by light and the circadian clock (Kaczorowski and Quail, 2003; Farré and Kay, 2007). There were five members in the PRR family in *Arabidopsis*, namely, TOC1 (PRR1), PRR3, PRR5, PRR7, and PRR9, and there was a close relationship between them. *TOC1* (*PRR1*) was the first PRR-like gene to be cloned and was the central regulator (Strayer et al., 2000). *PRR3* could modulate the stability of *TOC1*, yet both genes exhibited partially overlapped expression patterns (Para et al., 2007). *PRR5* could interact with *TOC1 in vitro* and *in vivo* through the N-terminus, and TOC1–PRR5 oligomerization enhanced the accumulation of TOC1 in the nucleus (Wang et al., 2010). Overexpression of *PRR5* could inhibit the expression of PRR7 and PRR9 while enhancing the expression of PRR3 and TOC1 (Sato et al., 2002). The gene PRR5/PRR7/PRR9 played an overlapping and distinctive role in the central oscillator and could repress the expression of *CIRCADIAN CLOCK ASSOCIATED1* (*CCA1*) and *LATE ELONGATED HYPOCOTYL* (*LHY*; Nakamichi et al., 2005, 2010). In rice and *Arabidopsis*, five PRR members showed a similar expression order, and five members started accumulating sequentially after dawn with 2–3 h intervals in the order of PRR9, PRR7, PRR5, PRR3, and PRR1 (Matsushika et al., 2000; Murakami et al., 2003).

After the identification of *PRR7* in *Arabidopsis*, homolog genes were gradually uncovered in many crops. *Ppd-H1* was the major determinant of barley photoperiod response, which enhanced the adaptation of spring-sown varieties (Turner et al., 2005). *OsPRR37* and *SbPRR37* were involved in regulating heading date and yield, and the loss-of-function genotype contributed to cultivation at a wide latitude area (Murphy et al., 2011; Koo et al., 2013). Studies have shown that genes *SbPRR37*, *OsPRR37*, *Ppd-H1*, and *PRR7* are all orthologous, possessing conserved functions in the photoperiodic pathway (Murphy et al., 2011; Gao et al., 2014). Here, we confirmed that overexpression of *SiPRR37* delayed the heading date in natural long-day conditions, which suggests that *SiPRR37* has a conservative function. The insertion of the transposon *Tc1-Marina* may be the result of adaptive selection.

The current research indicated that the PRR proteins also participate in abiotic stress regulation. Among the five PRR members in rice, *OsPRR73* could inhibit *OsHKT2;1* expression by binding the promoter and regulating the response to salt stress (Wei et al., 2021). In addition, *PRR5* interacts with *ABI5* and modulates expression to enhance ABA signal transduction during seed germination (Yang et al., 2021). In this study, we found that *SiPRR37* may be involved in the stress-related pathway through transcriptome analysis. Therefore, we speculated that *SiPRR37* also plays a role in the stress signal pathway, which requires further study.

## Data Availability Statement

The datasets presented in this study can be found in the NCBI Sequence Read Archive under accession number PRJNA797949. The names of the repository/repositories and accession number(s) can be found in the article/Supplementary Material.

## Author Contributions

LY, JW, and HuW designed the research. CL, JM, and GeW co-wrote the manuscript. HL, HaW, GuW, YJ, and YL performed the experiments and data analyses. GuiL, GuoL, and RC edited the article and were involved in many discussions. All authors contributed to the article and approved the submitted version.

## Funding

This work was supported by grants from the Beijing Municipal Natural Science Foundation (6202011), the National Key R&D Program of China (2018YFD1000706/2018YFD1000700), the Hebei Modern Seed Industry Innovation Team of Hybrid and Superior Quality Foxtail Millet (21326302D), the HAAFS Agriculture Science and Technology Innovation Project (2022KJCXZX-GZS-1), and the HAAFS Institute of Millet Crops Open Project of Grain Research (ZLSYS2020KF01).

## Conflict of Interest

The authors declare that the research was conducted in the absence of any commercial or financial relationships that could be construed as a potential conflict of interest.

## Publisher’s Note

All claims expressed in this article are solely those of the authors and do not necessarily represent those of their affiliated organizations, or those of the publisher, the editors and the reviewers. Any product that may be evaluated in this article, or claim that may be made by its manufacturer, is not guaranteed or endorsed by the publisher.

## Footnotes

^1^ https://phytozome-next.jgi.doe.gov

^2^ http://pfam.xfam.org/

^3^ http://us.expasy.org/tools/protparam.html

^4^ https://itol.embl.de/

^5^ http://bioinformatics.psb.ugent.be/webtools/plantcare/html/

^6^ https://www.wur.nl/en/show/MapChart-2.32.htm

^7^ http://pdgd.njau.edu.cn:8080

^8^ http://foxtail-millet.biocloud.net/page/tools/expressionVisualization

^9^ http://mapman.gabipd.org/web/guest/app/mercator
