# Supplementary material for: Exploring the SiCCT Gene Family and Its Role in Heading Date in Foxtail Millet
Source: Front Plant Sci. 2022 Jun 9;13:863298. doi: 10.3389/fpls.2022.863298 (PMC9218912; doi:10.3389/fpls.2022.863298)
Supplement: Supplementary file 1 [file Data_Sheet_1.PDF]

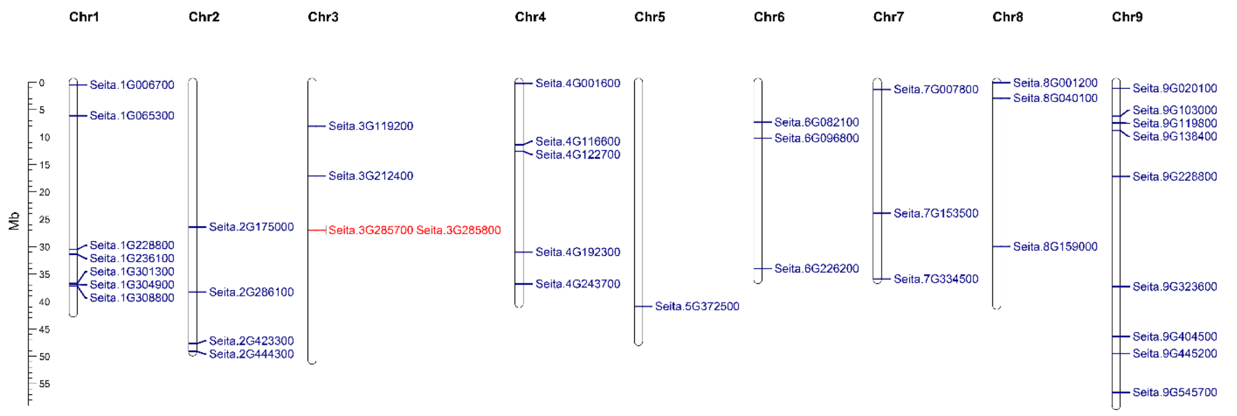

Supplementary Fig. S1 Chromosome localization of CCT family genes.
The CCT genes are denoted on the chromosomes in blue, and the tandemly duplicated genes are indicated in red.

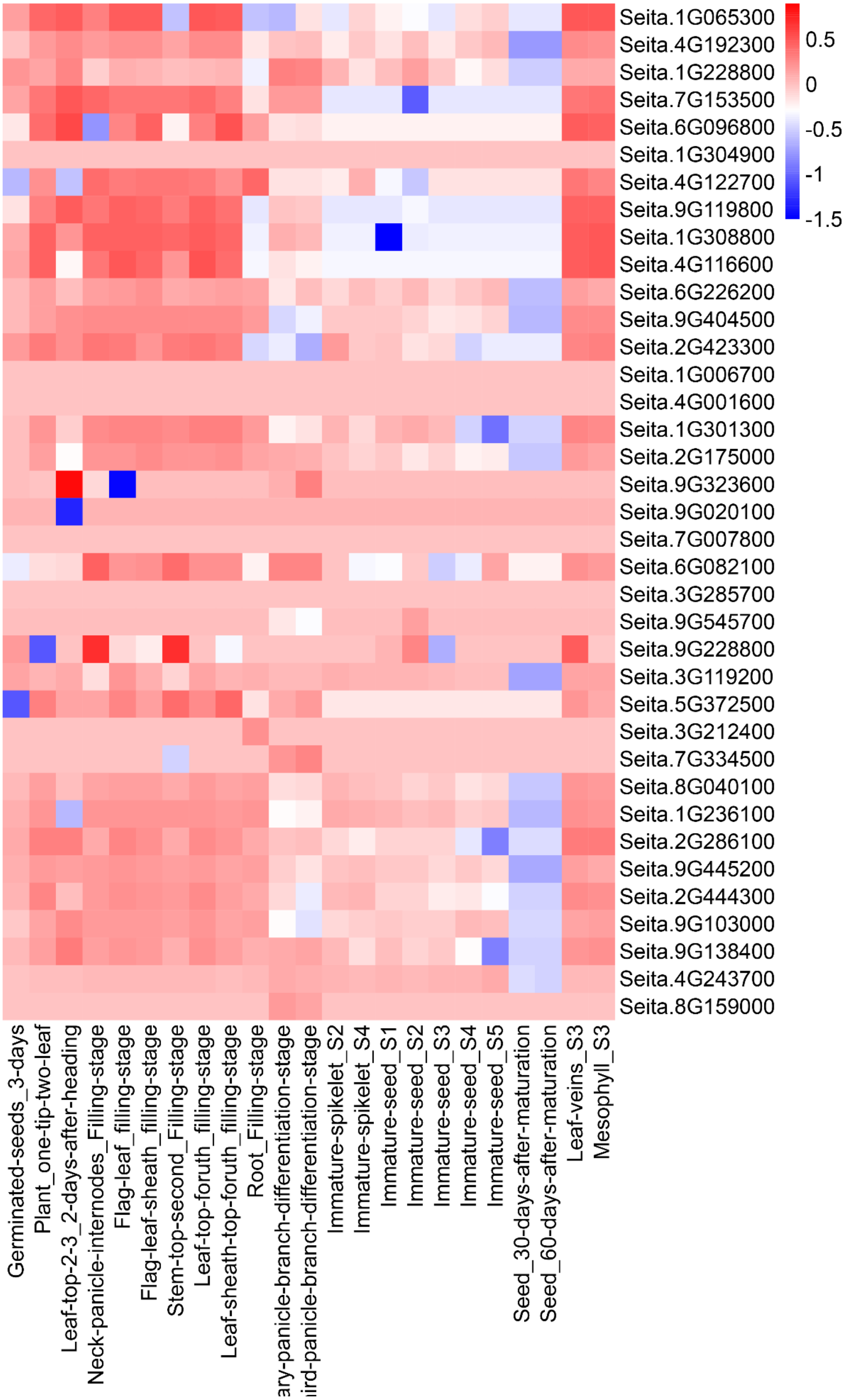

Supplementary Fig. S2 Heatmap of expression profiles of CCT genes in different tissues and at different growth periods. The FPKM values of CCT genes were downloaded from the multi-omics database for *Setaria italica* (MDSi), and the figure was shown in base 2 logarithmic form. The color represented relative expression levels from high (red) to low (blue).

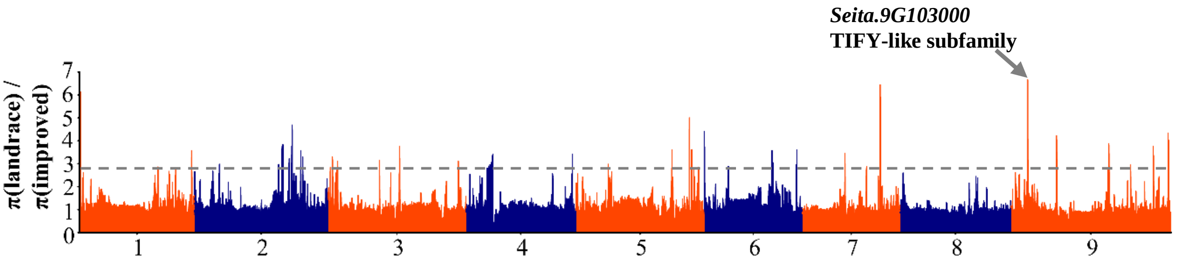

Supplementary Fig. S3 Whole-genome screening of selective sweeps in foxtail millet. The top 1% of $\pi_{landrace}/\pi_{improved\ cultivar}$ value was recognized as the threshold (the grey dashed line).

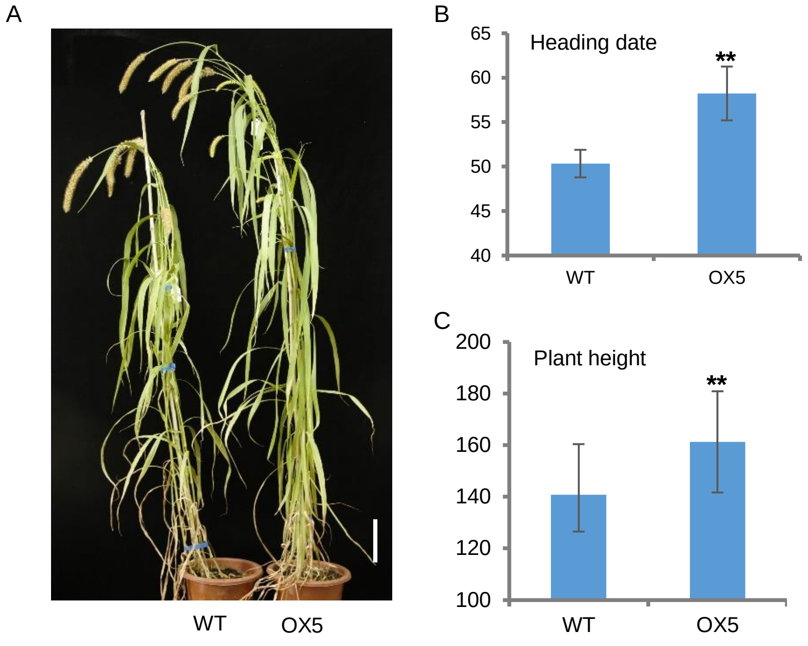

Supplementary Fig. S4 Phenotypic characterization of *SiPRR37* overexpression transgenic plants.
(A) Phenotype of transgenic line OX5 and wild type at the maturation stage grown in the greenhouse in 2020. Scale bar indicates 10cm.
(B) Statistical analysis of days to heading of transgenic line OX5 and wild type by Student's t-test. Error bars represent ± SD (n = 10). ** indicates P <0.01.
(C) Statistical analysis of plant height of transgenic line OX5 and wild type by Student's t-test. Error bars represent ± SD (n = 10). ** indicates P <0.01.

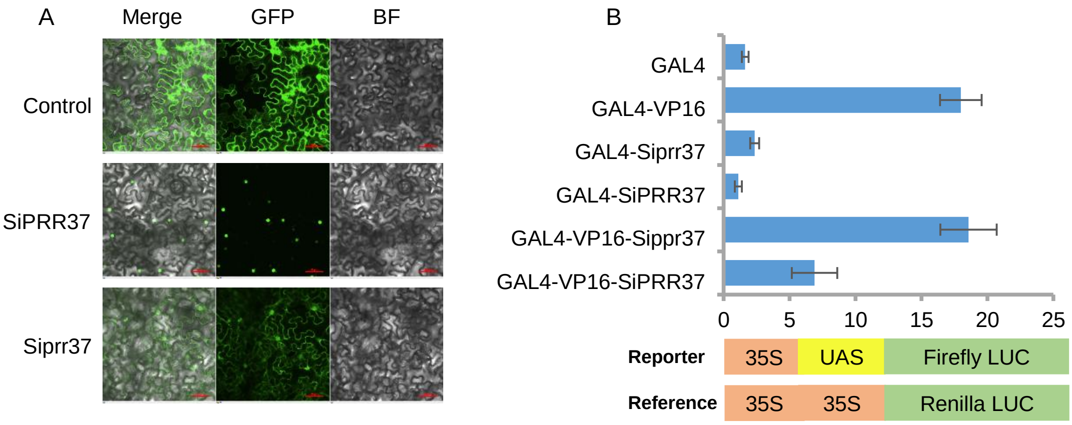

Supplementary Fig. S5 The subcellular localization and transcriptional activity analysis of *SiPRR37*.

(A) The subcellular localization of SiPRR37-GFP and Siprr37-GFP in tobacco.

(B) Transcriptional activity analysis of SiPRR37 and Siprr37 in *Arabidopsis* protoplast.
