# Supplementary material for: Exploring the SiCCT Gene Family and Its Role in Heading Date in Foxtail Millet
Source: Front Plant Sci. 2022 Jun 9;13:863298. doi: 10.3389/fpls.2022.863298 (PMC9218912; doi:10.3389/fpls.2022.863298)
Supplement: Supplementary file 2 [file Data_Sheet_2.PDF]

Supplementary Table S1 Characterization of 39 CCT genes identified in foxtail millet

| Gene ID | Chr | Start | End | Number of amino acid | Molecular Weight (kDa) | pI |
|---|---|---|---|---|---|---|
| Seita.1G006700 | 1 | 465291 | 467800 | 430 | 46.00 | 5.6 |
| Seita.1G065300 | 1 | 6104110 | 6106078 | 386 | 39.46 | 6.32 |
| Seita.1G228800 | 1 | 30543382 | 30544974 | 334 | 35.04 | 5.12 |
| Seita.1G236100 | 1 | 31446809 | 31449074 | 494 | 55.18 | 5.42 |
| Seita.1G301300 | 1 | 36700539 | 36705654 | 407 | 44.61 | 4.99 |
| Seita.1G304900 | 1 | 36911502 | 36917028 | 385 | 42.97 | 8.28 |
| Seita.1G308800 | 1 | 37159612 | 37161723 | 465 | 50.22 | 5.75 |
| Seita.2G175000 | 2 | 26390356 | 26396304 | 406 | 43.63 | 5.2 |
| Seita.2G286100 | 2 | 38248092 | 38252795 | 630 | 70.29 | 6.22 |
| Seita.2G423300 | 2 | 47693652 | 47695471 | 385 | 40.49 | 6.1 |
| Seita.2G444300 | 2 | 49119628 | 49133923 | 755 | 81.61 | 8.11 |
| Seita.3G119200 | 3 | 8016901 | 8021003 | 325 | 35.52 | 4.93 |
| Seita.3G212400 | 3 | 17135827 | 17137630 | 348 | 38.46 | 6 |
| Seita.3G285700 | 3 | 26980654 | 26983143 | 316 | 34.91 | 4.65 |
| Seita.3G285800 | 3 | 26999529 | 27002024 | 315 | 34.67 | 4.79 |
| Seita.4G001600 | 4 | 166471 | 167707 | 279 | 30.16 | 6.84 |
| Seita.4G116600 | 4 | 11409166 | 11411234 | 445 | 47.59 | 5.09 |
| Seita.4G122700 | 4 | 12554772 | 12557633 | 370 | 40.35 | 5.24 |
| Seita.4G192300 | 4 | 31034683 | 31036515 | 372 | 39.57 | 6.13 |
| Seita.4G243700 | 4 | 36749969 | 36754425 | 358 | 38.03 | 5.01 |
| Seita.5G372500 | 5 | 40855046 | 40859938 | 349 | 36.80 | 6.2 |
| Seita.6G082100 | 6 | 7334900 | 7337912 | 313 | 33.10 | 7.65 |
| Seita.6G096800 | 6 | 10227984 | 10228829 | 281 | 28.43 | 6.42 |
| Seita.6G226200 | 6 | 33985702 | 33990622 | 494 | 52.09 | 6.13 |
| Seita.7G007800 | 7 | 1292074 | 1294395 | 275 | 30.48 | 7.69 |
| Seita.7G153500 | 7 | 23921963 | 23923906 | 326 | 34.39 | 5.15 |
| Seita.7G334500 | 7 | 35892991 | 35893823 | 221 | 24.05 | 6.79 |
| Seita.8G001200 | 8 | 81134 | 82497 | 236 | 25.89 | 6.5 |
| Seita.8G040100 | 8 | 2905196 | 2909496 | 566 | 62.00 | 7.4 |
| Seita.8G159000 | 8 | 29964878 | 29966596 | 122 | 13.82 | 10.36 |
| Seita.9G020100 | 9 | 1056893 | 1059177 | 248 | 27.29 | 6.86 |
| Seita.9G103000 | 9 | 6233595 | 6237391 | 308 | 33.00 | 8.66 |
| Seita.9G119800 | 9 | 7441623 | 7443495 | 406 | 44.45 | 6.65 |
| Seita.9G138400 | 9 | 8770125 | 8774406 | 297 | 31.66 | 4.74 |
| Seita.9G228800 | 9 | 17246224 | 17248625 | 412 | 43.12 | 4.37 |
| Seita.9G323600 | 9 | 37297809 | 37302609 | 286 | 29.32 | 7.14 |
| Seita.9G404500 | 9 | 46372764 | 46376231 | 410 | 44.31 | 5.22 |
| Seita.9G445200 | 9 | 49505694 | 49513839 | 760 | 82.67 | 6.07 |
| Seita.9G545700 | 9 | 56607512 | 56611242 | 387 | 42.37 | 4.64 |

Supplementary Table S2 Conservative cis-elements statistics of CCT family genes

| Cis-regulatory elements | Core promoter sequences | Characteristics | Number |
|---|---|---|---|
| ABRE | GCAACGTGTC | abscisic acid responsiveness | 140 |
| ARE | AAACCA | anaerobic induction | 41 |
| AuxRR-core | GGTCCAT | auxin responsiveness | 13 |
| TGA-element | AACGAC | auxin-responsive element | 29 |
| circadian | CAAAGATATC | circadian control | 13 |
| TC-rich repeats | GTTTTCTTAC | defense and stress responsiveness | 9 |
| P-box | CCTTTTG | gibberellin-responsive element | 22 |
| GARE-motif | TCTGTTG | gibberellin-responsive element | 9 |
| TATC-box | TATCCCA | gibberellin-responsiveness | 5 |
| G-Box | CACGTT | light responsiveness | 158 |
| Sp1 | GGGCGG | light responsiveness | 54 |
| GT1-motif | GGTTAA | light responsiveness | 23 |
| ACE | GACACGTATG | light responsiveness | 3 |
| LTR | CCGAAA | low-temperature responsiveness | 33 |
| TGACG-motif | TGACG | MeJA-responsiveness | 84 |
| CGTCA-motif | CGTCA | MeJA-responsiveness | 84 |
| CAT-box | GCCACT | meristem expression | 31 |
| MBS | CAACTG | MYB binding site involved in drought-inducibility | 38 |
| MBSI | aaaAaaC(G/C)GTTA | MYB binding site involved in flavonoid biosynthetic genes regulation | 3 |
| MRE | AACCTAA | MYB binding site involved in light responsiveness | 11 |
| TCA-element | TCAGAAGAGG | salicylic acid responsiveness | 9 |
| RY-element | CATGCATG | seed-specific regulation | 9 |

Supplementary Table S3 List of segmentally duplicated CCT genes

| Type of duplications | Gene1 | Gene2 |
|---|---|---|
| Whole-genome duplications | Seita.1G304900 | Seita.4G122700 |
| | Seita.1G308800 | Seita.4G116600 |
| | Seita.1G065300 | Seita.4G192300 |
| | Seita.1G006700 | Seita.4G001600 |
| | Seita.1G228800 | Seita.7G153500 |
| | Seita.2G423300 | Seita.9G404500 |
| | Seita.2G444300 | Seita.9G445200 |
| | Seita.3G212400 | Seita.5G372500 |
| | Seita.7G334500 | Seita.8G001200 |
| | Seita.9G103000 | Seita.9G138400 |
| | Seita.9G228800 | Seita.9G545700 |
| Tandem duplication | Seita.3G285700 | Seita.3G285800 |
| Dispersed duplication | Seita.1G065300 | Seita.7G153500 |
| | Seita.1G228800 | Seita.4G192300 |
| | Seita.1G236100 | Seita.2G286100 |
| | Seita.1G301300 | Seita.9G404500 |
| | Seita.1G304900 | Seita.7G153500 |
| | Seita.1G308800 | Seita.9G119800 |
| | Seita.2G175000 | Seita.9G404500 |
| | Seita.2G286100 | Seita.9G445200 |
| | Seita.2G423300 | Seita.6G226200 |
| | Seita.2G444300 | Seita.9G231000 |
| | Seita.3G119200 | Seita.9G545700 |
| | Seita.3G212400 | Seita.8G001200 |
| | Seita.4G116600 | Seita.9G119800 |
| | Seita.4G122700 | Seita.7G153500 |
| | Seita.4G192300 | Seita.7G153500 |
| | Seita.4G243700 | Seita.9G103000 |
| | Seita.5G372500 | Seita.8G001200 |
| | Seita.6G082100 | Seita.9G119800 |
| | Seita.6G096800 | Seita.4G192300 |
| | Seita.7G007800 | Seita.9G020100 |
| | Seita.7G334500 | Seita.9G228800 |
| | Seita.8G001200 | Seita.9G228800 |
| | Seita.8G040100 | Seita.2G286100 |
| | Seita.9G020100 | Seita.9G323600 |
| | Seita.9G103000 | Seita.8G159000 |
| | Seita.9G138400 | Seita.8G159000 |
| | Seita.9G404500 | Seita.6G226200 |
| | Seita.9G445200 | Seita.9G231000 |

| | | |
|---|---|---|
| Transposed duplication | Seita.6G096800 | Seita.1G065300 |
| | Seita.2G175000 | Seita.1G301300 |
| | Seita.9G020100 | Seita.1G304900 |
| | Seita.1G236100 | Seita.2G444300 |
| | Seita.2G286100 | Seita.2G444300 |
| | Seita.6G082100 | Seita.4G116600 |
| | Seita.4G243700 | Seita.9G138400 |
| | Seita.3G119200 | Seita.9G228800 |

Supplementary Table S4 Missense mutations identified in CCT family genes based on haplotype of 312 accessions

| Chromosome | Position | Reference | SNP variation | Variation type | Gene | Subfamily |
|---|---|---|---|---|---|---|
| Chr1 | 466097 | A | G | missense variant | *Seita.1G006700* | COL-like |
| Chr1 | 6104593 | T | G | missense variant | *Seita.1G065300* | COL-like |
| Chr1 | 6104824 | A | G | missense variant | *Seita.1G065300* | COL-like |
| Chr1 | 6104836 | C | T | missense variant | *Seita.1G065300* | COL-like |
| Chr1 | 6105583 | G | T | missense variant | *Seita.1G065300* | COL-like |
| Chr1 | 6105584 | C | T | missense variant | *Seita.1G065300* | COL-like |
| Chr1 | 6105850 | C | T | missense variant | *Seita.1G065300* | COL-like |
| Chr1 | 30544153 | G | C | missense variant | *Seita.1G228800* | COL-like |
| Chr1 | 31446854 | G | A | missense variant | *Seita.1G236100* | PRR-like |
| Chr1 | 31448103 | G | A | missense variant | *Seita.1G236100* | PRR-like |
| Chr1 | 31448359 | G | A | missense variant | *Seita.1G236100* | PRR-like |
| Chr1 | 31448515 | G | A | missense variant | *Seita.1G236100* | PRR-like |
| Chr1 | 31448564 | G | T | missense variant | *Seita.1G236100* | PRR-like |
| Chr1 | 31448988 | G | A | missense variant | *Seita.1G236100* | PRR-like |
| Chr1 | 36702521 | G | A | missense variant | *Seita.1G301300* | COL-like |
| Chr1 | 36912439 | C | G | missense variant | *Seita.1G304900* | COL-like |
| Chr1 | 36912710 | T | C | missense variant | *Seita.1G304900* | COL-like |
| Chr1 | 36912785 | A | G | missense variant | *Seita.1G304900* | COL-like |
| Chr2 | 26392494 | C | T | missense variant | *Seita.2G175000* | COL-like |
| Chr2 | 26393749 | A | C | missense variant | *Seita.2G175000* | COL-like |
| Chr2 | 26393750 | C | T | missense variant | *Seita.2G175000* | COL-like |
| Chr2 | 38252662 | C | G | missense variant | *Seita.2G286100* | PRR-like |
| Chr2 | 49129307 | G | A | missense variant | *Seita.2G444300* | PRR-like |
| Chr2 | 49131728 | A | G | missense variant | *Seita.2G444300* | PRR-like |
| Chr2 | 49131911 | G | A | missense variant | *Seita.2G444300* | PRR-like |
| Chr2 | 49132060 | C | G | missense variant | *Seita.2G444300* | PRR-like |
| Chr3 | 17137179 | A | G | missense variant | *Seita.3G212400* | CMF-like |
| Chr4 | 166977 | A | C | missense variant | *Seita.4G001600* | COL-like |
| Chr4 | 167080 | T | C | missense variant | *Seita.4G001600* | COL-like |
| Chr4 | 12555331 | G | A | missense variant | *Seita.4G122700* | COL-like |
| Chr6 | 33986402 | T | A | missense variant | *Seita.6G226200* | COL-like |
| Chr6 | 33989522 | C | T | missense variant | *Seita.6G226200* | COL-like |
| Chr7 | 1293932 | C | T | missense variant | *Seita.7G007800* | CMF-like |
| Chr7 | 1294082 | C | T | missense variant | *Seita.7G007800* | CMF-like |
| Chr7 | 35893751 | T | C | missense variant | *Seita.7G334500* | CMF-like |
| Chr8 | 81655 | A | G | missense variant | *Seita.8G001200* | CMF-like |
| Chr8 | 29966550 | T | C | missense variant | *Seita.8G159000* | TIFY-like |
| Chr9 | 6233918 | A | G | missense variant | *Seita.9G103000* | TIFY-like |
| Chr9 | 8770334 | G | C | missense variant | *Seita.9G138400* | TIFY-like |

Supplementary Table S5 The CCT genes colocalizing with identified QTLs related to flowering time

| ID | Origional QTL number | Chr | Start | End | Genes colocalizing with QTLs | Subfamily | Note |
|---|---|---|---|---|---|---|---|
| QTL1 | qFT1.1 | 1 | 8485801 | 27361846 | | | Mauro-Herrera *et al.*, (2012) |
| QTL2 | qFT2.1 | 2 | 433141 | 776919 | | | |
| QTL3 | qFT2.2 | 2 | 35559584 | 39040231 | Seita.2G286100 | PRR | |
| QTL4 | qFT3.1 | 3 | 14549235 | 21409052 | Seita.3G212400 | CMF | |
| QTL5 | qFT3.2 | 3 | 25239463 | 41228836 | Seita.3G285700, Seita.3G285800 | CMF, CMF | |
| QTL6 | qFT4.1 | 4 | 4008980 | 30054438 | Seita.4G116600, Seita.4G122700 | COL, COL | |
| QTL7 | qFT4.2 | 4 | 37852525 | 38070151 | | | |
| QTL8 | qFT5.1 | 5 | 7584497 | 26646438 | | | |
| QTL9 | qFT5.2 | 5 | 34626198 | 36863070 | | | |
| QTL10 | qFT5.3 | 5 | 41852157 | 44912514 | | | |
| QTL11 | qFT6.1 | 6 | 5164626 | | | | |
| QTL12 | qFT6.2 | 6 | 6657522 | 7486973 | Seita.6G082100 | CMF | |
| QTL13 | qFT7.1 | 7 | 31381380 | 34013804 | | | |
| QTL14 | qFT8.1 | 8 | 1640952 | 8184010 | Seita.8G040100 | PRR | |
| QTL15 | qFT9.1 | 9 | 20627101 | 20103486 | | | |
| QTL16 | qFT9.2 | 9 | 34762993 | 36161328 | | | |
| QTL17 | qhd4-long | 4 | 10989324 | 12235376 | Seita.4G116600 | COL | Zhang *et al.*, (2017) |
| QTL18 | qhd6-short | 6 | 34487237 | 34561297 | Seita.6G082100, Seita.6G096800, Seita.6G226200 | CMF, COL, CMF | |
| QTL19 | | 2 | 48400000 | 49100000 | Seita.2G444300 | PRR | Doust *et al.*, (2017) |
| QTL20 | | 3 | 34800000 | 45500000 | | | |
| QTL21 | | 4 | 4100000 | 32800000 | Seita.4G116600, Seita.4G122700, Seita.4G192300 | COL, COL, COL | |
| QTL22 | | 5 | 21200000 | 27900000 | | | |
| QTL23 | | 5 | 31500000 | 41900000 | Seita.5G372500 | CMF | |
| QTL24 | | 7 | 31900000 | 34300000 | | | |
| QTL25 | | 8 | 1600000 | 4000000 | Seita.8G040100 | PRR | |
| QTL26 | | 8 | 2200000 | 9600000 | Seita.8G040100 | PRR | |
| QTL27 | | 9 | 22200000 | 36500000 | | | |

Supplementary Table S6 The CCT genes adjacent to known GWAS signals related to heading date

| Chr | Peak position | Enviroment | Genes adjacent to signal | Subfamily | Note |
|---|---|---|---|---|---|
| 1 | 37211711 | Beijing, Changzhi enviroment | Seita.1G308800 | COL | Jia *et al.*, (2013) |
| 1 | 19069050 | Beijing enviroment | | | |
| 2 | 49164136 | Beijing, Anyang enviroments | Seita.2G444300 | PRR | |
| 3 | 43398944 | Beijing enviroment | | | |
| 3 | 49110614 | Beijing enviroment | | | |
| 6 | 34194935 | Beijing enviroment | | | |
| 1 | 37215226 | Anyang enviroment | Seita.1G308800 | COL | |
| 1 | 31628684 | Anyang enviroment | | | |
| 6 | 35266551 | Anyang enviroment | | | |
| 2 | 3982183 | Anyang enviroment | | | |
| 6 | 23688848 | Anyang enviroment | | | |
| 6 | 19522914 | Anyang enviroment | | | |
| 8 | 28507351 | Changzhi enviroment | | | |
| 6 | 19542292 | Changzhi enviroment | | | |
| 3 | 2931864 | Changzhi enviroment | | | |
| 3 | 7268533 | Changzhi enviroment | | | |
| 9 | 1768436 | Changzhi enviroment | | | |
| 9 | 41964861 | Changzhi enviroment | | | |
| 2 | 2711177 | Sanya enviroment | | | |
| 4 | 6694176 | Sanya enviroment | | | |
| 3 | 7304512 | Sanya enviroment | | | |
| 7 | 21537506 | Sanya enviroment | | | |
| 2 | 4972834 | Sanya enviroment | | | |
| 9 | 52683820 | Sanya enviroment | | | |
| 4 | 7560381 | Sanya enviroment | | | |
| 8 | 56072 | Sanya enviroment | Seita.8G001200 | CMF | |
| 9 | 41354263 | Sanya enviroment | | | |
| 5 | 32143327 | Sanya enviroment | | | |
| 4 | 40078579 | Sanya enviroment | | | |
| 2 | 1196321 | Sanya enviroment | | | |
| 1 | 1703131 | Sanya enviroment | | | |
| 9 | 40287623 | Sanya enviroment | | | |
| 1 | 40127292 | Sanya enviroment | | | |
| 4 | 2109934 | Sanya enviroment | | | |
| 9 | 45552878 | Sanya enviroment | | | |
| 9 | 17024123 | Sanya enviroment | | | |
| 1 | 39418697 | Sanya enviroment | | | |
| 2 | 49087012 | Beijing enviroment | Seita.2G444300 | PRR | Li *et al.*, (2021) |

Supplementary Table S7 Information of primers in this study

| Primer name | Forward primer (5' to 3') | Reverse primer (5' to 3') | Expect Size (bp) | Aims |
|---|---|---|---|---|
| SiPRR37-cDNA | TATTCATTAACTCAAGACCTGCCT | TGGAGTCCAATGCAGCCAT | 2395 bp | SiPRR37 CDS cloning |
| SiPRR37-over | TTACTTCTGCACTAGGTACCATGGGAGGTACCCATCAGCAACCG | GAATTCCCGGGGATCCTCATCTATCTCCAGCTCCTTCCCA | 2268 bp | Overexpression vector construction |
| SiPRR37-GFP | CGGTCCCGGGGGATCCATGGGAGGTACCCATCAGCAACCG | TGCTCACCATGGATCCTCTATCTCCAGCTCCTTCCCA | 2265 bp | Subcellular localization |
| Siprr37-GFP | CGGTCCCGGGGGATCCATGGGAGGTACCCATCAGCAACCG | TGCTCACCATGGATCCGGGTTCCTTCTTCCTGTGAG | 345 bp | Subcellular localization |
| SiPRR37-GAL4BD | GCTCTAGAATGGGAGGTACCCATCAGCAACCG | CGGGATCCTCATCTATCTCCAGCTCCTTCCCA | 2268 bp | Transcriptional activity assay |
| Siprr37-GAL4BD | GCTCTAGAATGGGAGGTACCCATCAGCAACCG | CGGGATCCCTAGGGTTCCTTCTTCCTGTGAGA | 348 bp | Transcriptional activity assay |
| SiPRR37-VP16-GAL4BD | TATCGATACCGTCGACATGGGAGGTACCCATCAGCAACCG | CCCCCTCGAGGTCGACTCATCTATCTCCAGCTCCTTCCCA | 2265 bp | Transcriptional activity assay |
| Siprr37-VP16-GAL4BD | TATCGATACCGTCGACATGGGAGGTACCCATCAGCAACCG | CCCCCTCGAGGTCGACCTAGGGTTCCTTCTTCCTGTGAGA | 345 bp | Transcriptional activity assay |
| SiPRR37-RT | TGATGTCACCCTCAGCTATTAAGG | ATTCTCCCGGGACACATCAAAAT | 215 bp | RT-qPCR |
| SiACTIN | TATCGTTCAAACAGATTTACGGCCT | TAGAGAAGAAGTGACGAAGCCTTG | 186 bp | RT-qPCR |
| SiJMJ30 | ATCTGAGGACTTGTATCCACACAC | TAACCGAGAAACTTGTGGAGAGAG | 200 bp | RT-qPCR |
| SiRVE2 | GATGATCAGTCCCGTCAGGAAAT | CATCCGAGGAGCAAACTTTCTTTG | 151 bp | RT-qPCR |
| SiCOL9 | TGATTCCAGCCTTTGT | CCAGCAACTGAACC | 174 bp | RT-qPCR |

| | | | | |
|---|---|---|---|---|
| | ATTCCTGT | TTCAGGAC | | |
| SiPGM1 | TCGATTTGTTTTCACT GATGGGTC | TGAGCTTTGAAATA GACAATGCCA | 179 bp | RT-qPCR |
